# Gender-Specific Metabolomic Profiling of Obesity in Leptin-Deficient *ob*/*ob* Mice by ^1^H NMR Spectroscopy

**DOI:** 10.1371/journal.pone.0075998

**Published:** 2013-10-03

**Authors:** Eun-Young Won, Mi-Kyung Yoon, Sang-Woo Kim, Youngae Jung, Hyun-Whee Bae, Daeyoup Lee, Sung Goo Park, Chul-Ho Lee, Geum-Sook Hwang, Seung-Wook Chi

**Affiliations:** 1 Medical Proteomics Research Center, KRIBB, Daejeon, Republic of Korea; 2 Laboratory Animal Center, KRIBB, Daejeon, Republic of Korea; 3 Department of Biological Sciences, Korea Advanced Institute of Science and Technology, Daejeon, Republic of Korea; 4 Integrated Metabolomics Research Group, Seoul Center, Korea Basic Science Institute, Seoul, Republic of Korea; 5 Graduate School of Analytical Science and Technology, Chungnam National University, Daejeon, Republic of Korea; National Research Council of Italy, Italy

## Abstract

Despite the numerous metabolic studies on obesity, gender bias in obesity has rarely been investigated. Here, we report the metabolomic analysis of obesity by using leptin-deficient *ob*/*ob* mice based on the gender. Metabolomic analyses of urine and serum from *ob*/*ob* mice compared with those from C57BL/6J lean mice, based on the ^1^H NMR spectroscopy in combination with multivariate statistical analysis, revealed clear metabolic differences between obese and lean mice. We also identified 48 urine and 22 serum metabolites that were statistically significantly altered in obese mice compared to lean controls. These metabolites are involved in amino acid metabolism (leucine, alanine, ariginine, lysine, and methionine), tricarbocylic acid cycle and glucose metabolism (pyruvate, citrate, glycolate, acetoacetate, and acetone), lipid metabolism (cholesterol and carnitine), creatine metabolism (creatine and creatinine), and gut-microbiome-derived metabolism (choline, TMAO, hippurate, *p*-cresol, isobutyrate, 2-hydroxyisobutyrate, methylamine, and trigonelline). Notably, our metabolomic studies showed distinct gender variations. The obese male mice metabolism was specifically associated with insulin signaling, whereas the obese female mice metabolism was associated with lipid metabolism. Taken together, our study identifies the biomarker signature for obesity in *ob*/*ob* mice and provides biochemical insights into the metabolic alteration in obesity based on gender.

## Introduction

Once associated only with more developed countries, obesity is now prevalent in less developed countries, reaching global epidemic proportions across all genders and age groups [1]. Obesity results from excessive caloric intake compared with caloric consumption. The risks of obesity are associated with severe diseases such as noninsulin-dependent diabetes mellitus (NIDDM), cardiovascular diseases, hypertension, hypercholesterolemia, arthritis, asthma, and cancers [2]. Despite extensive studies, the physiological and biochemical basis of obesity remains largely unknown, as it is caused by multiple factors, such as genetic predisposition, environment, and lifestyle as well as complex interactions between these factors.

Among the known genetic factors, leptin, a key hormone regulating a balance between food intake and energy expenditure, first identified in 1994 [3], was highlighted as an important genetic component of human obesity [4], [5] Mutations in leptin or leptin receptor genes have been reported to result in human obesity [6]–[9]. In particular, leptin-deficiency was shown to be associated with severe early-onset obesity in a 9-year-old girl and her cousin [6] and leptin treatment reduced their body weights to the normal range [10]. The C57BL/6J-Lep^ob^/Lep^ob^ (hereafter referred to as *ob*/*ob*) mouse contains a spontaneous mutation in the *ob* gene encoding leptin, resulting in profound obesity, NIDDM, and phenotypes that resemble human obesity [3].

Metabolomics, analogous to proteomics and genomics, is now a widely utilized bio-analytical methodology in systems biology which employs nuclear magnetic resonance (NMR) spectroscopy or mass spectrometry to define small molecules present in biological samples in response to genetic modifications or physiological stimuli [11]. The NMR-based analysis platform, which contains a wealth of quantitative chemical information, in combination with multivariate statistical analysis, has been successfully utilized to yield information pertaining to the qualitative and quantitative alterations of small molecules following various stimuli across data sets. To date, metabolomics has been utilized for biomarker discovery [12] and understanding disease processes [13] and gut microbial-host metabolic interactions [14]. Since obesity is a systemic disorder involving metabolic changes, metabolic profilings of obesity have been performed by using various animal models, including *db*/*db* mice [12], Zucker rats [15]–[17], growth hormone mutant mice [18], and high fat diet (HFD)-induced mice [19]–[21]. Despite its importance in obese animal model, however, metabolomics analysis has rarely been performed on *ob*/*ob* mice.

Here, we report the metabolomic profiling of obesity using the *ob*/*ob* mice. Our ^1^H-NMR-based metabolomics analyses, in combination with multivariate statistical analysis, demonstrate not only significantly altered metabolism in obesity but also gender-specific metabolic changes in obesity.

## Materials and Methods

### Ethics Statement

All experimental procedures were approved by Institutional Animal Care and Use Committee of Korea Research Institute of Bioscience and Biotechnology, and performed in accordance with the NIH Guide for the Care and Use of Laboratory Animals (NIH Publication 1985, revised 1996).

### Animals

C57BL/6J (B6) and *ob*/*ob* mice were purchased from the Jackson Laboratory (Bar Harbor, ME) and housed in a humidity (60%)-, temperature (25±2°C)-, and light (12 h light/dark cycle)-controlled environment. The mice were fed commercial standard mouse chow diet (Teklad rodent diet 2018S, Harlan, Madison, WI) and water *ad libitum*. At 10 weeks of age, we collected 24 h-urine from the mice housed in metabolic cages (Tecniplast, Buguggiate, Italy) and serum from the same mice following overnight fasting. The samples were stored at −80°C until NMR analysis.

### Sample Preparation

After urine and serum samples were thawed at room temperature, they were centrifuged at 13,000 rpm for 10 min to remove the pellet. The 400 µL of resulting supernatant from urine sample was mixed with 0.2 M phosphate-buffered saline (PBS) (pH 7.0), 0.018% NaN_3_, 5 mM 4,4-dimethyl-4-silapentane-1-sulfonic acid (DSS), and 10% D_2_O to the final volume of 600 µL. For serum sample, 200 µL of resulting supernatant was mixed with 0.9% NaCl and 10% D_2_O to the final volume of 600 µL. The final samples were placed in a 5-mm NMR tube (Wilmad Lab Glass, Buena, NJ).

### ^1^H NMR Spectroscopy

NMR data were acquired on a 600 MHz Agilent NMR spectrometer (Agilent, Santa Clara, California, CA) equipped with a triple-resonance 5-mm HCN salt-tolerant cryoprobe at Korea Basic Science Institute. The NOESYPRESAT with the presaturation of water or water suppressed Carr–Purcell–Meiboom–Gill (CPMG) spin-echo pulse sequence was used to obtain ^1^H NMR spectra for urine or serum samples, respectively. The NOESYPRESAT spectra were collected with 64 transients into 67,568 data points using a spectral width of 10,000 Hz, relaxation delay of 2 s, and a mixing time of 100 ms. The CPMG spectra were collected with 128 transients into 67,568 data points using a spectral width of 10,000 Hz, and relaxation delay of 2 s.

### NMR Data Processing

NMR spectra were phased and baseline-corrected using TOPSPIN software (version 3.0, Bruker Biospin, Rheinstetten, Germany). All acquired NMR spectra were referenced to DSS at 0 ppm or lactate at 1.32 ppm for urine or serum sample, respectively. Spectra were then divided into 0.005 ppm bins excluding the spectral regions 4.70–5.05 ppm and 0.00–0.63 ppm, corresponding to water and DSS (for urine sample), respectively. After normalization of the spectra to the total spectral area to account for differences within the samples, the binning data were converted to the ASCII format, which were further imported into MATLAB (R2008a; MathWorks, Inc., Natick, MA). All spectra were aligned using the correlation optimized warping (COW) method [22] to reduce chemical shift variability across the spectra.

### Multivariate Statistical Analysis

The resulting binning data were Pareto-scaled and the targeted profiling data (as shown below) were unit variance (UV)-scaled, which were used for multivariate statistical analysis by using SIMCA-P (version 12.0, Umetrics, Umeå, Sweden). Principle components analysis (PCA), a classical unsupervised multivariate pattern recognition method, was employed to examine the intrinsic variation within a group and to assess the clustering behavior between groups. An orthogonal partial least-squares discriminant analysis (OPLS-DA), a supervised pattern recognition method, was further performed to maximize the variation between groups and to determine the variables that contribute to this variation. The quality of models was validated by determining R^2^ (goodness of fit parameter) and Q^2^ (goodness of prediction parameter) values [23].

### Targeted Metabolite Profiling

For the identification and quantification of metabolites, processed NMR spectra were imported into Chenomx software, a module of NMR Suite (version 7.1, Chenomx, Edmonton, Canada). The assignment of ambiguous peaks due to peaks overlap was confirmed by spiking standard compounds. The 600 MHz library from Chenomx was used for the identification of individual compounds. The internal reference standard DSS of a known concentration was used to determine their concentrations for urine samples. For serum samples, the intensities of the identified metabolites were used to determine their relative concentrations. The resulting concentration data sets of each metabolite were further used for multivariate statistical analyses including PCA and OPLS-DA, in addition to the binning input data, as described above.

### Statistical Analysis of Identified Metabolites

For the detection of statistical differences of the metabolite levels between lean and obese groups, nonparametric Mann-Whitney *t*-tests were performed using PRISM (version 5.0, GraphPad Software, Inc., La Jolla, CA) with 95% probability level (p<0.05).

### Pathway Analysis

For the metabolites that significantly changed in obesity with p<0.05, pathway analysis and metabolite set enrichment analysis (MESA) were performed using MetaboAnalyst, a web-based metabolomics data analysis software [24]. The concentrations of statistically significant metabolites that have p<0.05 in *t*-tests and VIP score >1 from OPLS-DA were used for quantitative pathway enrichment analysis. The “metabolic pathway-associated metabolite sets” was selected as metabolite set library. All input data were autoscaled.

## Results

### B6 and *ob*/*ob* Mice

The leptin-deficient *ob*/*ob* mouse was used as a genetically induced obesity mouse model and the B6 mouse, a commonly studied experimental model of metabolic diseases, was employed as a control. As expected, the *ob*/*ob* mice weighed two and three times as much as the B6 mice for male and female, respectively. The sample number and average body weight of male and female B6 and *ob*/*ob* mice are listed in Table 1.

**Table 1:** The number and body weight of the mice used in this study.

Model	Male			Female		
	Sample	N	Body weight	Sample	N	Body weight
B6	Urine	10	22±1 g	Urine	9	16±1 g
	Serum	10		Serum	10	
ob/ob	Urine	11	44±1 g	Urine	8	43±1 g
	Serum	11		Serum	8	

### ^1^H NMR Spectra of Urine and Serum Samples

**Figure 1:**
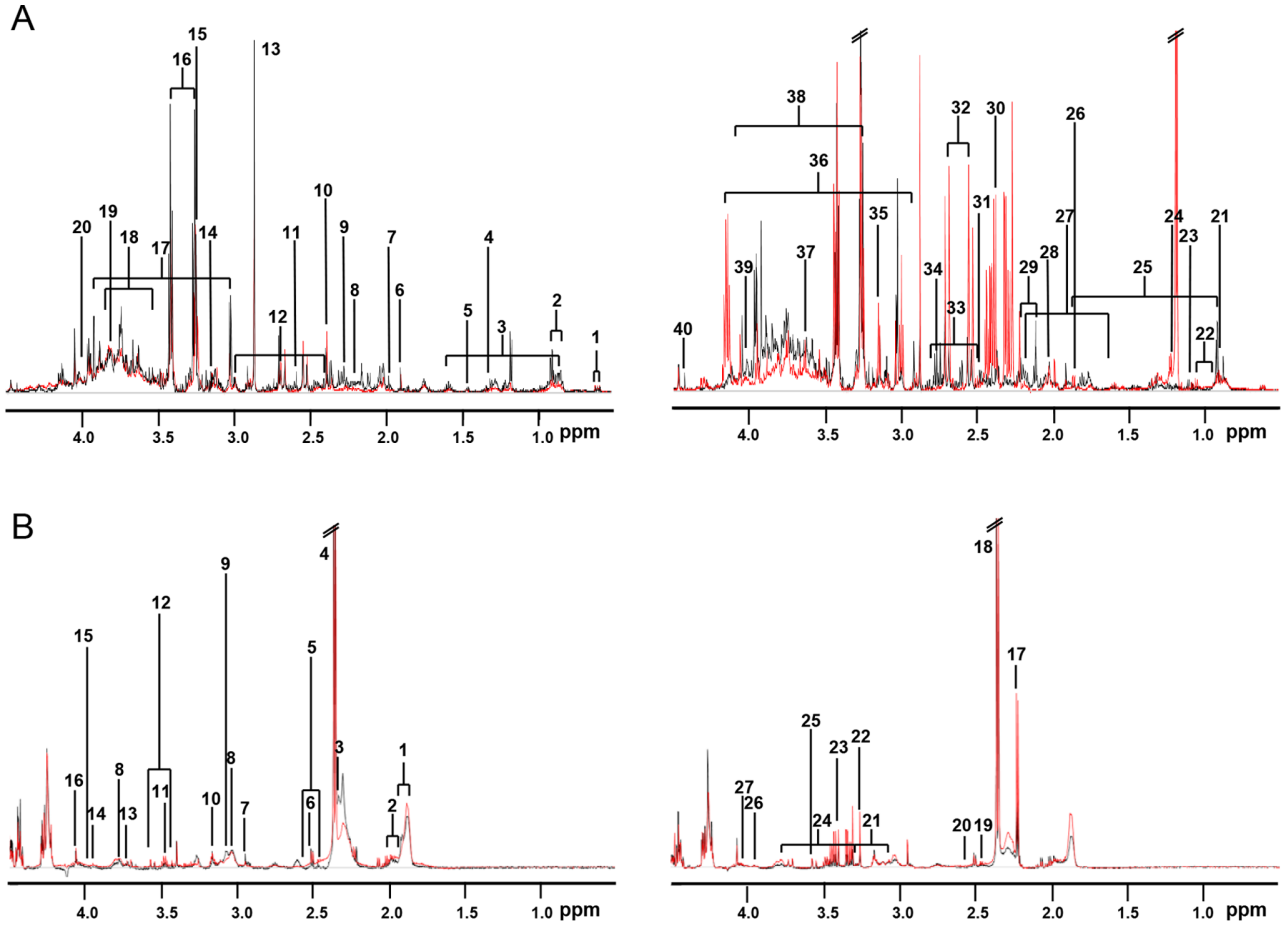
The overlaid representative ^1^H NMR spectra of urine (A) and serum (B) samples obtained from lean and obese mice. Black and red spectra are overlaid and shown for B6 mice and *ob*/*ob* mice, respectively. Samples from male and female mice are shown on the left and right, respectively. Each peak is labeled with the number that corresponds to the following metabolites. For urine samples (A), 1: pantothenate 2∶2-hydroxyisovalerate 3∶2-hydroxybutyrate 4∶2-hydroxyisobutyrate, lactate 5: alanine 6: acetate 7: N-acetyl aspartate 8: acetone 9: acetoacetate 10: succinate 11∶2-oxoisocaproate 12∶2-oxoglutarate 13: trimethylamine 14: methyl malonate 15: TMAO 16: taurine 17: creatinine 18: glycerol 19: guanidoacetate 20: pantothenate 21∶2-hydroxyvalerate 22: valine 23: isobutyrate 24: threonine 25: leucine 26: glutaric monomethyl ester 27: suberate 28: N-acetyal glutamate 29: methionine 30: pyruvate 31: methylamine 32: citrate 33∶5-aminolevulinate 34: dimethylamine 35: cis-aconitate 36: creatine 37: indole-3-acetate 38: choline 39: isopropanol 40: trigonelline. For serum samples (B), 1: VLDL/LDL cholesterol 2: isoleucine/leucine 3: valine 4: lactate 5: citrate 6: alanine 7: acetate 8: lysine 9: ornithine 10: TMAO 11: acetoacetate 12: glucose 13: ethylene glycol 14: alanine 15: glycolate 16: creatine 17: acetone 18: pyruvate 19: carnithine 20: methionine 21: phenylalanine 22: choline 23∶4-hydroxyphenylacetate 24: arginine 25: glycine 26: creatine 27: serine.

The representative ^1^H NMR spectra of urine and serum samples were overlaid for obese and lean mice (Figure S1). The clear spectral differences between obese and lean samples were mostly observed at the up-field region between 0 and 4 ppm (Figure 1), which contains information about the aliphatic groups of metabolites. Through the visual inspection of the NMR spectra, more intense peaks were observed for obese mice samples than lean ones, which was even more obvious for female than male and for urine than serum. This suggests potential metabolic changes in obese groups, when compared to lean controls, and gender-specific variations in obese groups.

Next, we identified and quantified 71 urine and 29 serum metabolites using the 600 MHz library within the Chenomx software. For identification of unambiguous peaks, spiking experiments were performed using several standard compounds. The concentrations of the identified metabolites were determined with respect to the internal standard DSS of a known concentration or lactate for urine and serum sample, respectively.

### Multivariate Statistical Analysis

In order to investigate whether there are statistically significant metabolic differences between B6 and *ob*/*ob* mice, we carried out multivariate statistical analysis using concentrations of the identified metabolites derived from targeted profiling. The PCA, an unsupervised multivariate pattern recognition method, does not show clear discrimination between B6 and *ob*/*ob* mice for urine samples, while it does for serum samples (Figure S2, left panels). Based on the NMR spectra showing the gender differences between B6 and *ob*/*ob* mice particularly for urine samples (Figure 1), we hypothesized that the separation between B6 and *ob*/*ob* mice was hindered by gender variation. As expected, a gender-specific PCA showed a better separation between B6 and *ob*/*ob* mice, as indicated by increased R^2^ (goodness of fit parameter) and Q^2^ (goodness of prediction parameter) values [23] (Figure S2, middle and right panels). Such effect was more obvious for the urine than serum samples. For urine samples, there is a separation between obese and lean subjects only when analyzed based on gender, whereas for serum samples, the separation that exists for all genders becomes better when analyzed based on gender. Although similar results were obtained when spectral binning data were used as an input (data not shown), artifacts can arise from the integration of input bins. For this reason, we used metabolic concentration data for further analysis.

**Figure 2:**
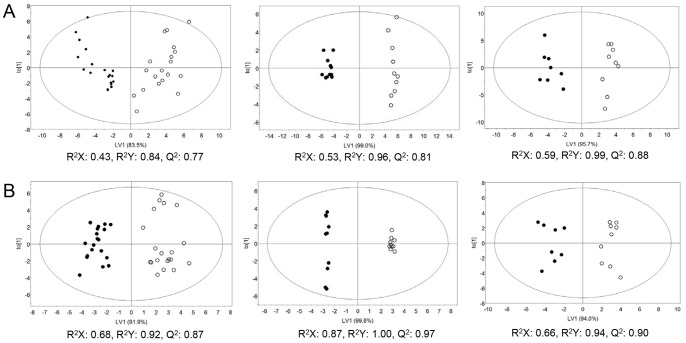
Orthogonal partial least-squares discriminant analysis (OPLS-DA) score plots obtained from the ^1^H NMR spectra of urine (A) and serum (B) samples from lean and obese mice. Data for all (left panels), male (middle panels), and female (right panels) are shown. Closed and open circles represent obese and lean mice, respectively.

To maximize the differences between the groups and determine the variables that contribute to discrimination, the OPLS-DA, a supervised pattern recognition method, was further employed for the same set of metabolic concentration data (Figure 2). The OPLS-DA model was validated using R^2^ and Q^2^ values [23], as shown in Figure 2. Although the OPLS-DA showed distinct separation between lean and obese mice regardless of gender (Figure 2, left panels), a gender-specific analysis provided an even better clustering between groups (Figure 2, middle and right panels). This suggests that the metabolic changes occur in obese mice compared to lean controls and such metabolic changes are dependent on gender.

**Figure 3:**
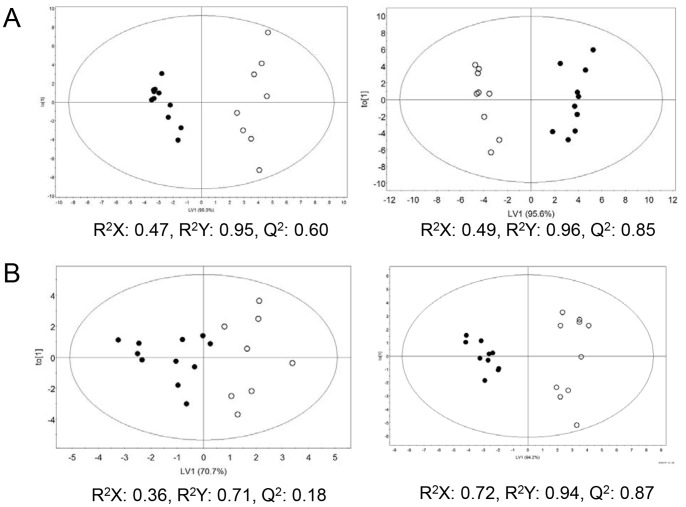
OPLS-DA score plots derived from the ^1^H NMR spectra of urine (A) and serum (B) samples from lean and obese mice based on gender. Data for obese (left panels) and lean (right panels) are demonstrated. Closed and open circles represent male and female mice, respectively.

Furthermore, to confirm the gender-specific metabolic changes in obesity, an OPLS-DA was also performed based on gender (Figure 3). Indeed, OPLS-DA of urine and serum samples from obese groups showed clear differences between males and females (Figure 3, left panels). Again, such differences were more obvious for urine than serum sample. However, gender variation was also observed for urine and serum samples from lean controls (Figure 3, right panels), indicating intrinsic metabolic variations between male and female. Thus, the gender variation observed in obesity is likely due to the intrinsic metabolic differences between males and females. Taken together, our multivariate statistical analyses indicate statistically significant metabolic differences between obese and lean mice, which can be further differentiated by gender.

### Identification of Biomarker for Obesity

We generated variable importance in project (VIP) plots from the OPLS-DA with a threshold of 1.0 (Figure S3) to identify the metabolites that significantly contribute to the clustering between obese and lean groups. The metabolites with VIP score >1, considered to be strong contributors, include 2-oxoisocaproate, creatine, leucine, 2-hydroxybutyrate, methionine, 2-hydroxyvalerate, suberate, alanine, creatinine, methylamine, trigonelline, choline, glutaric acid monomethyl ester (MME), hippurate, *p*-cresol, valine, N-acetylglutamate, guanidoacetate, phenylalanine, isopropanol, tyrosine, 5-aminolevulinate, allantoin, 3,5-dibromotyrosine, threonine, isobutyrate, 2-hydroxyisovalerate, and nicotinamide N-oxide for urine sample and arginine, creatine, lysine, very low-density lipoprotein (VLDL)/low-density lipoprotein (LDL) cholesterol, ornithine, glycolate, alanine, carnitine, trimethylamine N-oxide (TMAO), and pyruvate for serum sample (listed from high to low VIP scores).

For the concentrations of the identified metabolites, Mann-Whitney *t*-test was further employed with 95% probability level (p<0.05) to select the metabolites that were significantly altered in *ob*/*ob* mice, compared to lean mice. Using this *t*-test together with VIP score from OPLS-DA, we were able to select 28 out of 71 urine metabolites and 10 out of 29 serum metabolites for all obese mice including males and females, 37 and 22 urine metabolites from males and females, respectively, and 11 and 15 serum metabolites from male and female mice, respectively, all with 95% probability level (p<0.05) and VIP score >1. They are categorized and listed in Tables 2 and 3. Interestingly, most of the significantly altered metabolites are down-regulated in *ob*/*ob* mice, compared to B6 mice (Tables 2 and 3). This is probably because *ob*/*ob* mice are hypometabolic due to reduced circulating levels of thyrotropin and thyroid hormones. [25] The up-regulated metabolites include acetoacetate, acetone, citrate, fumarate, 2-oxoglutarate, succinate, trimethylamine (TMA), and 3-hydroxybutyrate for urine sample only from obese females and acetoacetate, acetone, succinate, carnitine, VLDL/LDL cholesterol, and TMAO for serum from obese mice, many of which are involved in tricarboxylic acid (TCA) cycle and lipid metabolism (Tables 2 and 3).

**Table 2:** Summary of significant urinary metabolites differentiating between ob/ob and B6 mice.

Pathway	Metabolite	All		Male		Female	
		Change^a^	VIP	Change^a^	VIP	Change^a^	VIP
Amino acid metabolism	Alanine	DOWN***	1.59	DOWN***	1.35	DOWN**	1.57
	5-Aminolevulinate	DOWN**	1.14	DOWN***	1.26	UP	0.08
	Guanidinoacetate	DOWN***	1.23	DOWN***	1.13	DOWN	0.80
	2-Hydroxybutyrate	DOWN***	1.65	DOWN***	1.46	DOWN*	1.43
	2-Hydroxyisovalerate	DOWN*	1.05	DOWN	0.07	DOWN	1.76
	3-Hydroxykynurenine	DOWN*	0.71	DOWN***	1.32	UP	0.43
	Isopropanol	DOWN***	1.21	DOWN***	1.16	DOWN	0.61
	Leucine	DOWN***	1.75	DOWN***	1.34	DOWN***	2.03
	Methionine	DOWN***	1.65	DOWN***	1.47	DOWN*	1.24
	Methylmalonate	DOWN	0.67	DOWN***	1.47	UP	0.57
	N-acetyl aspartate	DOWN*	0.91	DOWN**	1.07	DOWN	0.22
	N-acetyl glutamate	DOWN***	1.28	DOWN***	1.15	DOWN	0.82
	2-Oxoisocaproate	DOWN***	1.82	DOWN***	1.29	DOWN**	1.87
	Phenylalanine	DOWN***	1.22	DOWN***	1.25	DOWN*	1.22
	Threonine	DOWN**	1.09	DOWN***	1.33	UP	0.61
	Tryptophan	DOWN*	0.92	DOWN***	1.17	UP	0.20
	Tyrosine	DOWN**	1.16	DOWN***	1.14	DOWN	0.57
	Urea	DOWN	0.38	DOWN	0.35	DOWN*	1.31
	Valine	DOWN***	1.29	DOWN***	1.47	DOWN	0.60
TCA cycle and glucose metabolism	Acetate	DOWN	0.38	DOWN	0.14	DOWN**	1.47
	Acetoacetate	UP*	0.84	UP	0.51	UP*	1.31
	Acetone	UP*	0.78	DOWN	0.04	UP*	1.39
	Citrate	UP**	0.97	UP	0.22	UP**	1.59
	Fumarate	UP	0.61	UP	0.11	UP*	1.12
	2-oxoglutarate	UP*	0.76	UP	0.22	UP*	1.21
	Pyruvate	DOWN	0.38	DOWN***	1.18	UP	0.74
	Succinate	UP	0.27	UP	0.66	UP*	1.37
Lipid metabolism	Glycerol	DOWN***	0.91	DOWN***	1.16	UP	0.53
	Taurine	DOWN	0.64	UP	0.15	DOWN*	1.32
Creatine metabolism	Creatine	DOWN***	1.79	DOWN***	1.28	DOWN***	1.77
	Creatine phosphate	DOWN*	0.77	DOWN***	1.13	UP	0.85
	Creatinine	DOWN***	1.59	DOWN***	1.40	DOWN*	1.26
Gut microbiome-derived metabolism	Choline	DOWN***	1.45	DOWN***	1.20	DOWN**	1.61
	Dimethyamine	DOWN	0.48	DOWN**	1.05	UP	0.42
	Hippurate	DOWN***	1.38	DOWN***	1.19	DOWN	1.02
	2-Hydroxyisobutyrate	DOWN**	0.97	DOWN***	1.32	UP	0.51
	Isobutyrate	DOWN**	1.09	DOWN***	1.34	DOWN	0.84
	Methylamine	DOWN***	1.56	DOWN***	1.15	DOWN**	1.58
	p-Cresol	DOWN***	1.37	DOWN***	1.34	DOWN	0.65
	TMA^b^	DOWN	0.41	DOWN***	1.09	UP***	1.73
	Trigonelline	DOWN***	1.49	DOWN***	1.33	DOWN	0.98
Others^c^	Allantoin	DOWN**	1.11	DOWN***	1.19	DOWN	0.38
	3,5-Dibromotyrosine	DOWN**	1.11	DOWN***	1.23	UP	0.16
	Glutaric acid MME^d^	DOWN***	1.43	DOWN***	1.32	DOWN	0.82
	3-Hydroxybutyrate	UP	0.73	DOWN	0.42	UP**	1.46
	2-Hydroxyvalerate	DOWN***	1.60	DOWN***	1.57	DOWN**	0.76
	Nicotinamide N-oxide	DOWN**	1.00	DOWN***	1.28	UP	0.51
	Suberate	DOWN***	1.59	DOWN***	1.25	DOWN*	1.38
Total no.^e^	48	28		37		22	

All the metabolites that have p<0.05 in *t*-test and VIP score >1 from OPLS-DA are shown for all, male, and/or female. The p values of the individual metabolic concentration in *t*-test are symbolized as *, **, and ***, indicating p<0.05, p<0.01, and p<0.005, respectively and the metabolites with no asterisk are less significant, having p>0.05.

a Direction of change is shown for obese mice compared to lean controls.

b Abbreviated for trimethylamine.

c Other metabolites that do not belong to any of the metabolic categories listed above.

d Abbreviated for monomethyl ester.

e Total number of significant metabolites with VIP score >1 and p<0.05 for all, male, and female.

**Table 3:** Summary of significant serum metabolites differentiating between ob/ob and B6 mice.

Pathway	Metabolite	All		Male		Female	
		Change^a^	VIP	Change^a^	VIP	Change^a^	VIP
Amino acidmetabolism	Alanine	DOWN***	1.18	DOWN	0.54	DOWN***	1.50
	Arginine	DOWN***	1.86	DOWN***	1.82	DOWN***	1.58
	Glycine	DOWN*	0.73	UP	0.35	DOWN**	1.24
	Isoleucine	DOWN*	0.73	UP	0.36	DOWN**	1.05
	Lysine	DOWN***	1.59	DOWN***	1.80	DOWN**	1.07
	Methionine	DOWN*	0.70	UP	0.36	DOWN***	1.22
	Ornithine	DOWN***	1.44	DOWN***	1.76	DOWN*	0.86
	Serine	DOWN**	0.98	DOWN	0.49	DOWN**	1.13
TCA cycle and glucosemetabolism	Acetoacetate	UP*	0.69	UP**	1.07	UP	0.72
	Acetone	UP**	0.91	UP	0.77	UP**	1.00
	Citrate	DOWN*	0.65	UP	0.15	DOWN**	1.05
	Glucose	DOWN	0.53	DOWN**	1.15	DOWN	0.03
	Glycolate	DOWN***	1.19	DOWN*	0.88	DOWN**	1.22
	Lactate	DOWN*	0.74	DOWN	0.22	DOWN**	1.15
	Pyruvate	DOWN***	1.03	DOWN**	1.17	DOWN	0.71
	Succinate	UP	0.61	UP**	1.17	UP	0.56
Lipid metabolism	Carnitine	UP***	1.07	UP**	1.15	UP*	0.83
	VLDL/LDL cholesterol	UP***	1.50	UP***	1.38	UP***	1.33
Creatine metabolism	Creatine	DOWN***	1.73	DOWN***	1.78	DOWN***	1.38
Gut microbiomemetabolism	Choline	DOWN**	0.96	DOWN	0.32	DOWN**	1.15
	TMAO	UP***	1.05	UP***	1.35	UP	0.57
Others^b^	Ethylene glycol	DOWN*	0.80	DOWN	0.01	DOWN**	1.06
Total no.^c^	22	10		11		15	

All the metabolites that have p<0.05 in *t*-test and VIP score >1 from OPLS-DA are shown for all, male, and/or female. The p values of the individual metabolic concentration in *t*-test are symbolized as *, **, and ***, indicating p<0.05, p<0.01, and p<0.005, respectively and the metabolites with no asterisk are less significant, having p>0.05.

a Direction of change is shown for obese mice compared to lean controls.

b Other metabolites that do not belong to any of the metabolic categories listed above.

c Total number of significant metabolites with VIP score >1 and p<0.05 for all, male, and female.

To identify the metabolites that contribute to the clustering between genders, we generated VIP plots from the OPLS-DA with a threshold of 1.0, based on the genders (Figure S4). After removing the metabolites that contribute to the clustering between genders in urine and serum samples from lean mice, the metabolites that have unique association with gender variation in obese mice are identified and listed in Table 4. The VIP score and/or p value of some metabolites were significantly altered in obese mice, while the relatively high levels of other metabolites, based on gender, were reversed in obese male mice compared to lean mice. The latter includes urea, succinate, pyruvate, methyl malonate, 2-hydroxyvalerate, glycerol, nicotinamide N-oxide, and threonine for urine sample and alanine and isoleucine for serum sample and the former includes the remainders. Interestingly, 72% of urine metabolites from obese mice with VIP score >1 have a unique association with gender, i.e. 28% of them already exist in lean mice differentiating the genders, whereas 60% of serum metabolites from obese mice have a unique association with genders. This is consistent with the previous statistical analysis that the gender variations were more distinct for the urine than serum samples (Figures 1, 2, and S2).

**Table 4:** Summary of significant metabolites differentiating between males and females in ob/ob mice.

Metabolite	VIP score	High in^a^
Urine		
Urea	1.80	M***
1-Methyl nicotinamide	1.76	F***
Acetone	1.60	F***
3-Hydroxybutyrate	1.60	F***
Succinate	1.57	F***
Pyruvate	1.49	F**
Methyl malonate	1.42	F*
Acetoacetate	1.39	F*
2-Hydroxyvalerate	1.36	F*
Glucarate	1.35	F*
Glycerol	1.34	F*
Nicotinamide N-oxide	1.26	F*
Threonine	1.20	F*
Serum		
Alanine	1.88	M*
Isoleucine	1.83	M*
Arginine	1.71	F*

This analysis employed three parameters, VIP score from OPLS-DA, p value in *t*-test, and relatively high metabolic concentrations between the genders, to identify the significant metabolites differentiating between the genders in obese group. If the VIP score and/or p value of a metabolite was altered significantly or its relatively high concentration was reversed between the genders, among the significant metabolites with VIP score >1 and p<0.05, the metabolite was listed above. For the VIP plot for all samples, see Figure S3.

a This indicates whether the metabolic concentration is relatively high in male or female. Also, the p values of the individual metabolic concentration in *t*-test are symbolized as *, **, and ***, indicating p<0.05, p<0.01, and p<0.005, respectively.

### Pathway Analysis

**Figure 4:**
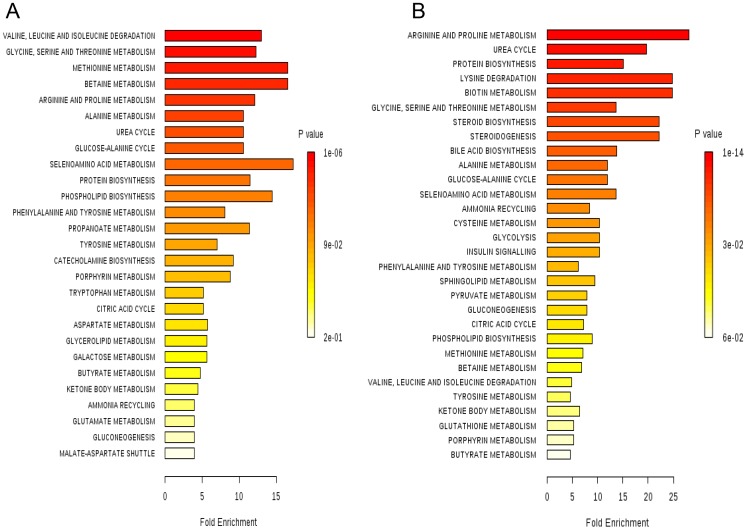
Summary of pathway enrichment analysis of urine (A) and serum (B) samples. Each pathway is ranked from low (red) to high (white) Holm p-value, corresponding to the most and the least significant values, respectively.

To gain insight into the metabolic mechanism of obesity, metabolic pathways of the significantly altered metabolites were analyzed using the “pathway analysis” module within the MetaboAnalyst software [24]. We identified a total of 27 and 31 distinct metabolic pathways that were significantly altered in the urine (Table S1) and serum (Table S2) samples from obese group, respectively. Next, we performed metabolite set enrichment analysis (MSEA) for the same set of significant metabolic concentration data to test if there arebiologically significant groups of metabolites that are enriched in obesity. Using this method, we identified a number of potentially affected metabolic pathways in urine and serum from obese mice and found that many of them were involved in amino acid metabolism (Figure 4). This was expected because many of the significantly altered metabolites with obesity were amino acids and their degradation products, as listed in Tables 2 and 3. In addition to amino acid metabolism, other affected pathwayswere involved in lipid-related metabolisms, which included betain metabolism, phospholipid biosynthesis in both urine and serum and steroid biosynthesis, steroidogenesis, and sphingolipid metabolism in serum. This is consistent with the fact that obesity is characterized by excessive fat deposits in tissue [26]. The enriched pathways in amino acid metabolism and lipid metabolism are related to biotin metabolism in serum, as biotin is involved in fatty acid synthesis and amino acid catabolism by acting as a prosthetic group for biotin-dependent carboxylases [27]. Urea cycle, another perturbed pathway in both urine and serum, is related to creatine metabolism [28].

**Figure 5:**
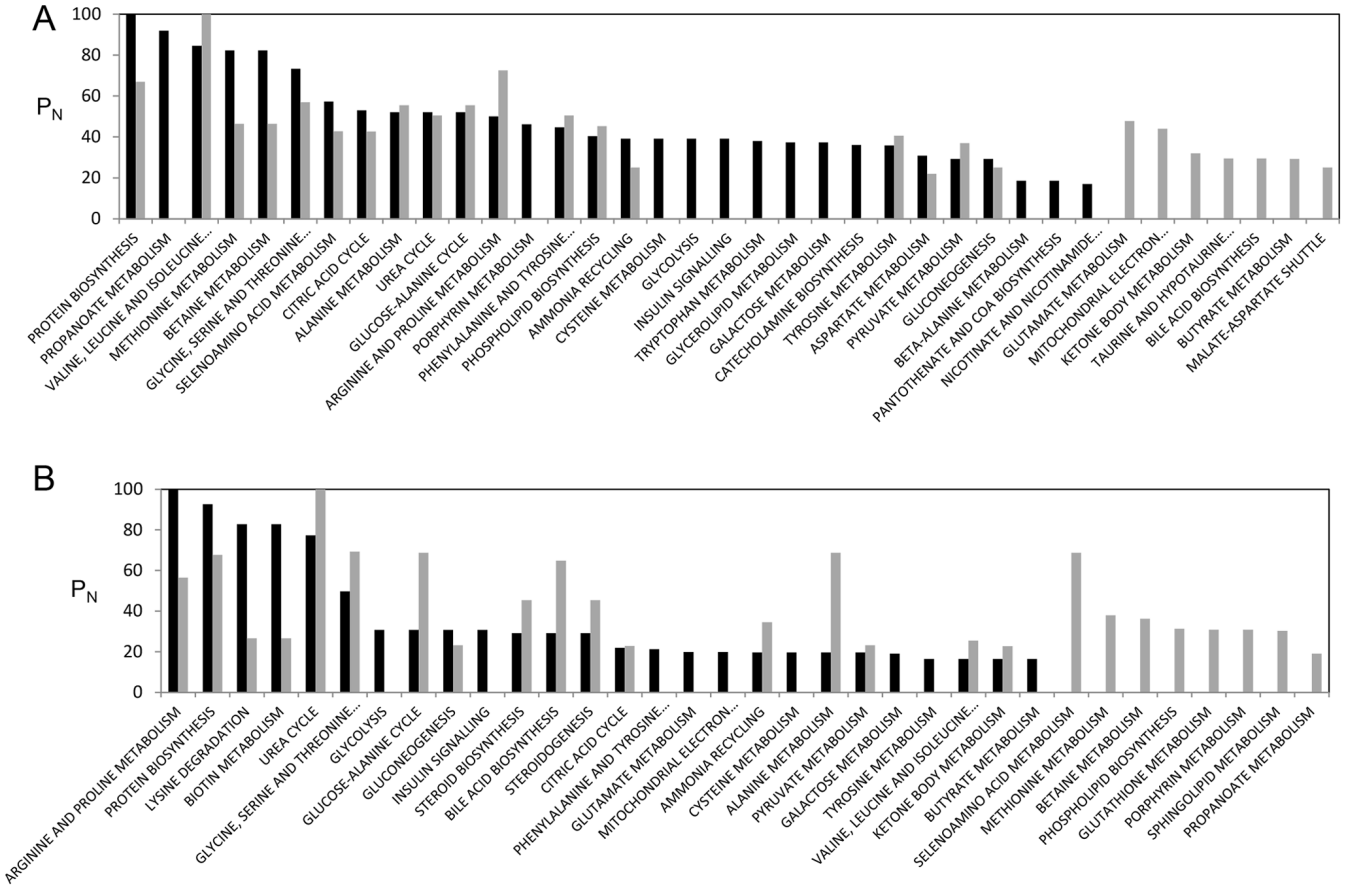
Summary of the relatively enriched pathways urine (A) and serum (B) samples based on gender. The log values of – (Holm p-value) are normalized (P_N_) within the same gender to enable the direct comparison of the relatively enriched metabolic pathways between male and female. The pathways for male and female are shown as black and gray bars, respectively.

For the analysis of gender-specific metabolic pathway changes, we performed MSEA based on gender (Figure S5). The input data for this analysis were concentrations of 37 and 22 urine metabolites from male and female mice, respectively, and 11 and 15 serum metabolites from male and female mice, which were significantly altered in obesity with VIP score >1 and p<0.05 (Tables 2 and 3). We computed the normalized log values of – (Holm p-value) (P_N_) to enable direct comparison of the relative impact of individual pathways within the same gender with high P_N_ having the high impact. The comparison of P_N_ values of MSEA for each sample (Figure 5) demonstrated that amino acid metabolisms that showed distinct differentiation between obese and lean mice (Figure 4, Tables 2 and 3), did not significantly differ between male and female mice. This suggests that amino acid metabolites are significant biomarkers of obesity regardless of gender. The metabolic pathways enriched only in male were associated with diabetes-related pathways, including insulin signaling, glycolysis and galactose metabolism. In contrast, female-specific metabolic features included lipid-related metabolisms, such as betaine metabolism, sphingolipid metabolism, and phospholipid biosynthesis. Relatively large P_N_ values were observed for steroid biosynthesis, steroidogenesis, and sphingolipid metabolism in the females.

## Discussion

Our metabolomic profiling of obesity in *ob*/*ob* mice reveals significant gender variations in obesity as well as distinct metabolic differences between obese and lean mice, as monitored by ^1^H NMR spectroscopy. Despite extensive metabolomics studies on obesity, gender has been rarely considered to be an important factor in analyzing metabolomics data in obesity to date. Here, we report the systemic metabolomic profiling of obesity based on gender. Despite its importance in the obesity animal model, *ob*/*ob* mice have rarely been utilized for the metabolomics study of obesity. To our knowledge, only one report using this mouse model has been reported, but was only limited to analysis of fat content of fatty pancreas [29]. Similar to *ob*/*ob* mice, leptin receptor-deficient *db*/*db* mice have been used in several metabolomic studies as a model of NIDDM. [12], [29], [30] Although the several phenotypes are shared by both mouse models (e.g. obesity and insulin resistance), *ob*/*ob* mouse model differs from *db*/*db* model such that leptin-deficiency was shown to be specifically associated with severe early-onset obesity in obese humans [6]. The metabolic alterations and gender variations observed in *ob/ob* mice, compared with B6 mice, are discussed below.

### Metabolic Alterations in Obesity

#### Amino acid metabolism

The most significant metabolites differentiating between obese and lean mice are related to amino acid metabolism (Figure 4 and Tables 2 and 3). In particular, we observed significantly depleted level of leucine (VIP score of 1.75 for all; p<0.001) in urine samples from obese mice compared with those from lean controls (Table 2). The depletion of leucine is consistent throughout our data. The depletion of 2-oxoisocaproate, a degradation product from leucine by leucine transaminase, and pyruvate and alanine, the metabolites related to leucine catabolism and elevation of acetoacetate, an end product of leucine in obese mice, compared to lean mice (Tables 2 and 3), also indicate the depletion of leucine. Leucine plays key roles in modulation of insulin signaling [31], protein synthesis in skeletal muscle [32] and production of alanine/glutamine in skeletal muscle [33], [34] via mammalian target of rapamycin (mTOR) pathway [35], [36]. The reduced levels of leucine were observed in HFD-induced obese Sprague-Dawley rat [37], HFD-B6 mice [19], [20], and NIDDM individuals [38], which are consistent with our results (Table 2). Importantly, the roles of leucine in the regulation of muscle protein and insulin signaling seem to be consistent features throughout our data. Since leucine stimulates muscle protein synthesis and inhibits protein degradation in skeletal muscle and liver [32], [39], significant depletion of leucine in obesity is related to skeletal muscle atrophy. Indeed, the urinary excretion of creatinine, degraded from creatine in muscle in proportion to muscle mass [40], was significantly reduced in obese mice compared to lean mice (VIP score of 1.59; p<0.001, Table 2), suggesting skeletal muscle atrophy. Consistent with this, obesity has been shown to be associated with skeletal muscle atrophy [41]. Regarding the role of leucine in insulin signaling, down-regulated glycolysis as indicated by depletion of pyruvate and lactate, a feature of insulin resistance, was observed in obese mice (Table 3). In addition, significantly reduced levels of alanine in both urine and serum from obese mice (Tables 2 and 3) are related to depletion of leucine because leucine is the major nitrogen source for alanine synthesis in muscle. [33], [34] Previously, leucine has been suggested to be used to treat obesity [35]. Even leucine alone has been shown to reduce insulin resistance, improve glucose tolerance, and restore metabolic abnormalities in HFD-induced mice [42], and reduce insulin resistance in *db*/*db* mice [43]. Taken together, leucine can be used as a significant biomarker for obesity.

Other significantly altered amino acids in urine and/or serum samples from *ob*/*ob* mice include arginine, lysine, methionine, glycine, serine, threonine, and tryptophan (Tables 2 and 3). In particular, the significantly lowered level of arginine in serum sample from obese mice (VIP score of 1.86; p<0.001, Table 3) is in agreement with the anti-obesity effect of arginine [44], [45]. Several lines of evidences have shown that dietary arginine supplementation reduced obesity in genetically obese rats, HFD-induced rats, and obese humans by promoting the oxidation of glucose and fatty acids as well as by decreasing *de novo* synthesis of glucose and triacylglycerols [45]–[47]. In addition, the depletion of lysine and methionine suggests increased insulin resistance. Consistent with previous metabolomic profiling of HFD-mice [20], we observed significantly lowered levels of lysine in serum from obese mice compared to lean controls (VIP score of 1.59; p<0.001, Table 3). Lysine is involved in energy metabolism, implying the perturbations in insulin resistance [20]. The concentration of methionine, a key metabolite in lipid homeostasis and insulin sensitivity [19], was significantly reduced in *ob*/*ob* mice compared to lean controls (VIP score of 1.65; p<0.001 for urine sample, Table 2), which is consistent with the previous report on HFD-B6 mice [19]. Another altered amino acid glycine was also depleted in obese samples (Table 3), which is consistent with previous reports [16], [21], [48], [49]. The depletion of glycine was likely due to the decreased levels of serine and pyruvate in serum sample from obese mice (Table 3), because glycine can be generated from serine, which is, in turn, derived from pyruvate [40]. In addition, it is related to the lowered level of urea in urine sample from female obese mice (Table 2) because glycine is an important precursor for urea biosynthesis [40].

#### TCA cycle and glucose metabolism

The TCA cycle is a series of biochemical reactions that are utilized by all aerobic organisms to produce energy. Acetate, in the form of acetyl coenzyme A (acetyl-CoA), derived from pyruvate via glycolysis, enters the TCA cycle to produce citrate and other metabolites under aerobic conditions. Our metabolomics analysis of urine demonstrates elevated levels of several TCA cycle metabolites including acetoacetate, acetone, citrate, fumarate, 2-oxoglutarate, and succinate particularly for obese females (Tables 2), suggesting the up-regulation of TCA cycle in obese female mice. In particular, the level of citrate was elevated in the urine from the obese female mice, whereas depleted in the serum from the obese female mice (Tables 2 and 3). The citrate is endogenously generated from fatty acid and glucose metabolism and regulated by insulin and glucose levels [50]. The increased levels of citrate were observed in alloxan diabetic rats [51] and HFD-induced obese animals [20], [21] due to hyperglycaemia and insulin resistance [20], while the lowered levels were seen in other obese animals [17], [18], [21]. Despite this discrepancy regarding the levels of citrate, alterations of citrate levels suggest the disturbances in insulin or glucose levels in obesity. In contrast to the TCA cycle, depleted levels of pyruvate and acetate (Tables 2 and 3), corresponding to the entry points into the TCA cycle, were observed, suggesting down-regulated glycolysis in obesity. In addition, the level of lactate, a product of pyruvate under anaerobic condition, was lowered in serum from obese mice (Table 2), again suggesting down-regulated glycolysis under anaerobic condition.

#### Lipid metabolism

Fat deposits in tissue are a significant feature of obesity and are often associated with elevated levels of free fatty acids or cholesterol in the blood [26]. We observed significant elevation of VLDL/LDL cholesterol in serum from obese mice (VIP score of 1.50; p<0.001, Table 3), which is consistent with the previous studies [15], [21], [26]. Another key metabolite identified in our study is carnitine. Carnitine plays key roles in fatty acid metabolism by participating in the transport of fatty acids from the cytosol into the mitochondria to produce energy via β-oxidation [52], [53]. Due to the direct association of carnitine levels with β-oxidation, depleted levels of carnitine was previously observed in liver tissue of HFD-induced mice [54] and blood of obese humans [55]. However, elevated carnitine levels were also observed in the serum of HFD-induced mice [26], [54]. In this study, we observed significantly high levels of carnitine in both obese male and female serum, compared to lean controls. Taken together, whether increased or decreased, the significant changes in carnitine level indicate the disturbance in energy metabolism with obesity.

#### Creatine metabolism

**Figure 6:**
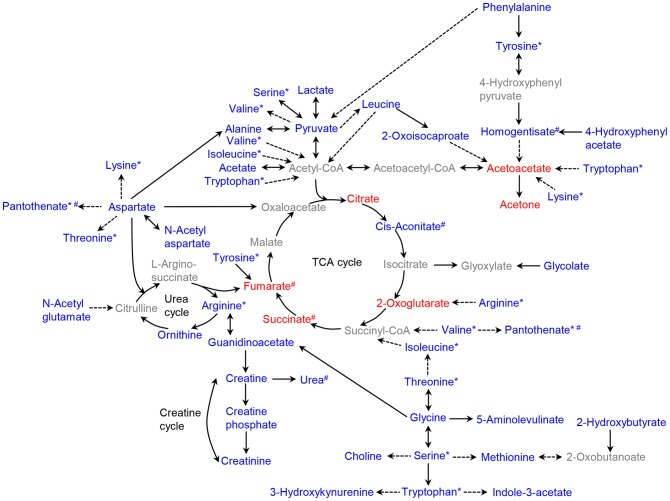
Major metabolic pathways for the metabolites that are significantly altered in urine and serum from *ob*/*ob* mice compared to lean controls, observed by ^1^H NMR spectroscopy. The pathways were analyzed on the basis of the KEGG pathway database (http://www.genome.jp/kegg/pathway.html). The levels of the identified metabolites that were elevated and depleted in obese mice, compared to lean mice, are color coded as red and blue, respectively, and the metabolites in gray were not identified in our study. Direction of change is shown for all obese mice including males and females. For the direction of change based on gender, see Tables 2 and 3. The metabolites with asterisk are shown several times in this diagram to avoid the complexity of the pathways. The metabolites with sharp indicate the significant ones in male or female group only. Solid and dashed arrows indicate the single and multiple steps involved, respectively, between two metabolites.

Creatine and creatine phosphate serve as a rapid source of high-energy phosphate by the reaction of creatine phosphate with ADP to produce ATP. They are broken down into creatinine in muscle [56]. Since creatinine is usually produced at levels proportional to muscle mass [40] and obesity is associated with skeletal muscle atrophy [41], depletion of creatine, creatine phosphate, and creatinine is expected. Indeed, our metabolomic analysis showed a significant depletion of urinary excretion of creatine, creatine phosphate, and creatinine and serum concentration of creatine in obese mice compared to lean controls (Tables 2 and 3). In addition, depletion of creatine is also associated with the reduced levels of arginine and glycine in obese serum (Table 3), because creatine is biosynthesized from these amino acids [56]. Major metabolic pathway networks, including amino acid metabolism, TCA cycle, glucose metabolism, and creatine metabolism, are summarized and illustrated in Figure 6.

#### Gut microbiome-derived metabolism

Gut microbiome has been reported to be able to influence susceptibility to obesity by altering the efficiency of energy harvest from the diet [57]. Although the two dominant bacterial divisions, the Bacteroidetes and the Firmicutes, are found in a majority of human and mouse gut, their relative abundance is altered by obesity [58]. In obese mice and humans, the number of Firmicutes increased, whereas the number of Bacteroidetes decreased compared to lean individuals. Thus, the identified metabolites and their related pathways, originating from gut microflora, are often altered in obese human and mice. In particular, the pathways from choline to TMA and TMAO are indicative of the gut microbiota status [40], [59]. Our metabolomics analysis showed that the levels of choline and methylamine were significantly decreased in obese urine and serum, whereas the level of TMAO was significantly increased in obese serum, compared to lean controls (Tables 2 and 3). Consistent with our results, methylamine levels were reported to be attenuated in obese phenotype of *db*/*db* mice [12]. The elevation of serum TMAO was also observed in GHR mutant obese mice [18], whereas decreased TMAO was reported in serum and urine from HFD-induced obese mice or Zucker rats [16], [19], [20]. An inverse relationship of microbiome-originated metabolites might be due to variations in the mouse strain utilized [60]. Recently, Koeth *et al.* reported that TMAO can be produced from dietary carnitine by intestinal microbiota, resulting in the acceleration of atherosclerosis in mice [61]. Consistent with this finding, our analysis shows the elevation of carnitine and TMAO in serum from obese mice compared to lean controls (Table 3), suggesting the gut-microbiota-mediated metabolism of TMAO.

Another microbiota-derived metabolite, hippurate, is generated from the conjugation of glycine and benzoic acid that is produced from aromatic amino acids by gut microbes and excreted into the urine [62]. The significance of the urinary excretion of hippurate has been considered for obesity and diabetes [63]. The urinary level of hippurate in our study was significantly decreased, compared to lean controls (p<0.005; VIP score of 1.38, Table 2), which is consistent with previous studies using other obese animal models [16], [20], [64], [65] as well as obese humans [66]. A significantly decreased urinary excretion of *p*-cresol, generated from tyrosine by gut microflora [67], was observed in the obese mice (p<0.005; VIP score of 1.37, Table 2). In addition, depletion of gut microflora-originated short-chain fatty acid isobutyrate in obese urine (Table 2) suggests decreased bacterial fermentation of leucine and valine, resulting in the alteration of host energy metabolism [68]. Urinary excretion of 2-hydroxyisobutyrate, a compound derived from the microbial degradation of dietary protein, has been shown to be associated with some members of the gut microflora, for example, *F. prausnitzii* [69]. The urinary level of trigonelline, produced via the conversion of S-adenosylmethionine (SAM) to S-adenosylhomocysteine (SAH) by gut microflora [70], was also significantly decreased in obese mice, when compared to lean controls (p<0.005; VIP score of 1.49, Table 2). This suggested that SAM was consumed and thus depleted in the trans-sulfuration pathway to produce glutathione that was depleted in obesity [66], [70].

### Gender Variations in Obesity

Our metabolomics analysis showed significant gender variations in obesity. Although our OPLS-DA analyses showed distinct separation between lean and obese subjects, such discrimination was even more clear when analyzed based on gender (Figure 2). In addition, this gender variation was more obvious for urine than serum samples and for female than male, as observed in the NMR spectra (Figure 1) and OPLS-DA analyses based on obesity (Figure 2) and gender (Figure 3).

Pathway enrichment analysis based on gender (Figure 5) showed that female-specific pathways are mainly related to lipid metabolism. One of the metabolites that significantly contribute to the metabolic variations in female obese group is taurine. Taurine plays key roles in conjugation of cholesterol and bile acids, antioxidation, osmoregulation, and calcium signaling [71]. Previous reports showed that the levels of taurine were decreased [16], [19] and that taurine supplementation decreased serum lipids and body weight in obese human [72]. In our analysis, taurine was significantly depleted in urine from obese females, whereas no significant alteration was observed in obese males (Table 2). Female-specific lipid metabolism may arise from the female sex hormone, estrogen. Previous studies reported the strong positive correlation of estrogen with lipid synthesis and the excretion of citrate [73], [74] because estrogen increases the availability of fatty acids [75] and urinary citrate excretion [74]. In addition to lipid-related metabolism, the urinary excretion of citrate was significantly increased in obese female urine with high VIP score (1.59), whereas only marginal increase was observed in obese male urine with low VIP score (0.22) (Table 2). Other female-specific metabolites are urea and glycine. The urinary level of urea was significantly lowered in obese females (p<0.05; VIP score of 1.31, Table 2), whereas only marginally lowered in obese males (p>0.05; VIP score of 0.35, Table 2). In addition, urea had the highest VIP score (1.80) from the OPLS-DA based on gender (Table 4). The depletion of urea in obese females is related to significant decrease in the level of glycine in serum from obese females (Table 3) because glycine is an important precursor for urea biosynthesis [40]. Contrary to the level of glycine in obese males, it was significantly decreased in obese females (p<0.01; VIP score of 1.24, Table 3).

In contrast to females, males are specifically associated with diabetes-related pathways, including insulin signaling, glycolysis and galactose metabolism (Figure 5). Some of the metabolites that significantly contributed to the metabolic variations in male obese group include pyruvate in urine (Table 2) and glucose in serum (Table 3) from obese male mice. These metabolites were significantly decreased compared to obese females (Tables 2 and 3) and contribute to the separation between males and females in obese mice (Table 4). The depletion of urinary pyruvate in obese males suggests down-regulation of glycolysis and thus an insulin resistance. Although some previous studies indicated that there were no gender difference in diabetes and obesity, other reports suggested that the male sex hormone was a risk factor in the development of diabetes due to the changes in the hormone levels in diabetes [76]–[79]. In particular, significantly low levels of testosterone have been observed in obese humans [80] and diabetic men [76], [78]. Furthermore, hypoandrogenism has been proposed as an early biomarker for the disturbances in insulin and glucose metabolism [77]. Thus, the alterations in insulin signaling unique to obese males in our study may arise from such depletion of testosterone.

These gender-specific metabolic differences are thought to originate from intrinsic metabolic differences between males and females because there are clear metabolic differences between genders even in lean controls (Figure 3). The intrinsic gender-specific metabolic differences have been also reported in normal mice [81] and humans [82], suggesting the fundamental metabolic differences between genders. Thus, it is likely that the fundamental metabolic differences between genders, regardless of metabolic abnormality, led to the gender-specific metabolic variation in obesity. Despite the significant effect of gender on the metabolism of obesity, only one report has been previously published to our knowledge, which showed the gender variation in obese Zucker rats [64]. Based on our results, we suggest that the gender be considered in metabolomics profiling of obesity.

The risks and damages caused by obesity is not only limited to individual health problems; it also leads to social economic problems by increasing health care cost [83]. Given that obesity is a multifactorial problem, early diagnosis and treatment are critical to improve personal health and prevent the resulting economic burden. In this study, our ^1^H-NMR-based metabolomic profiling of obesity using leptin-deficient *ob*/*ob* mouse has shown metabolic changes and gender variations in obesity. Future work is needed in human samples to validate the biomarker signatures of obesity identified in our study and to characterize leptin-deficient obesity, which can be further used for anti-obesity drug development.

## Supporting Information

Figure S1 **Full overlaid spectra of representative ^1^H NMR spectra of urine sample from lean and obese mice (** **Figure 1A** **).** Samples from male and female mice are shown on the top and bottom, respectively. Black and red spectra are overlaid and shown for lean mice and ob/ob mice, respectively. Since there are almost no peaks in the upfield region of the spectra for serum samples and all the peaks are shown in Figure 1B, full view of the spectra are not shown here.
(TIF)

Figure S2 **PCA score plots obtained from the ^1^H NMR spectra of urine (A) and serum (B) samples from lean and obese mice.** All the mice including males and females, male mice, and female mice are shown on the left, middle, and right, respectively.
(TIF)

Figure S3 **Variable importance in the projection (VIP) plots of lean** ***vs*** **. obese mice obtained from OPLS-DA with a threshold of 1.0.** All the mice including males and females, males, and females are shown on the top, middle, and down, respectively, for urine (A) and serum (B) samples.
(TIF)

Figure S4 **Variable importance in the projection (VIP) plots of male** ***vs*** **. female mice obtained from OPLS-DA with a threshold of 1.0.** The urine (A) and serum (B) samples from the obese (top) and lean (down) mice are shown.
(TIF)

Figure S5 **Pathway enrichment analysis of urine (A) and serum (B) samples.** The 47 and 23 urine metabolites from male and female mice, respectively, and 13 and 19 serum metabolites from male and female mice, respectively, all with p<0.05 in Mann-Whitney *t*-tests, were used for quantitative pathway enrichment analysis.
(TIF)

Table S1 **Summary of significantly altered urine metabolites and the related metabolic pathways in ob/ob mice in detail.**
(DOCX)

Table S2 **Summary of significantly altered serum metabolites and the related metabolic pathways in ob/ob mice in detail.**
(DOCX)
