# Supplementary figures and images for: Gender-Specific Metabolomic Profiling of Obesity in Leptin-Deficient ob/ob Mice by 1H NMR Spectroscopy
Source: PLoS One. 2013 Oct 3;8(10):e75998. doi: 10.1371/journal.pone.0075998 (PMC3789719; doi:10.1371/journal.pone.0075998)

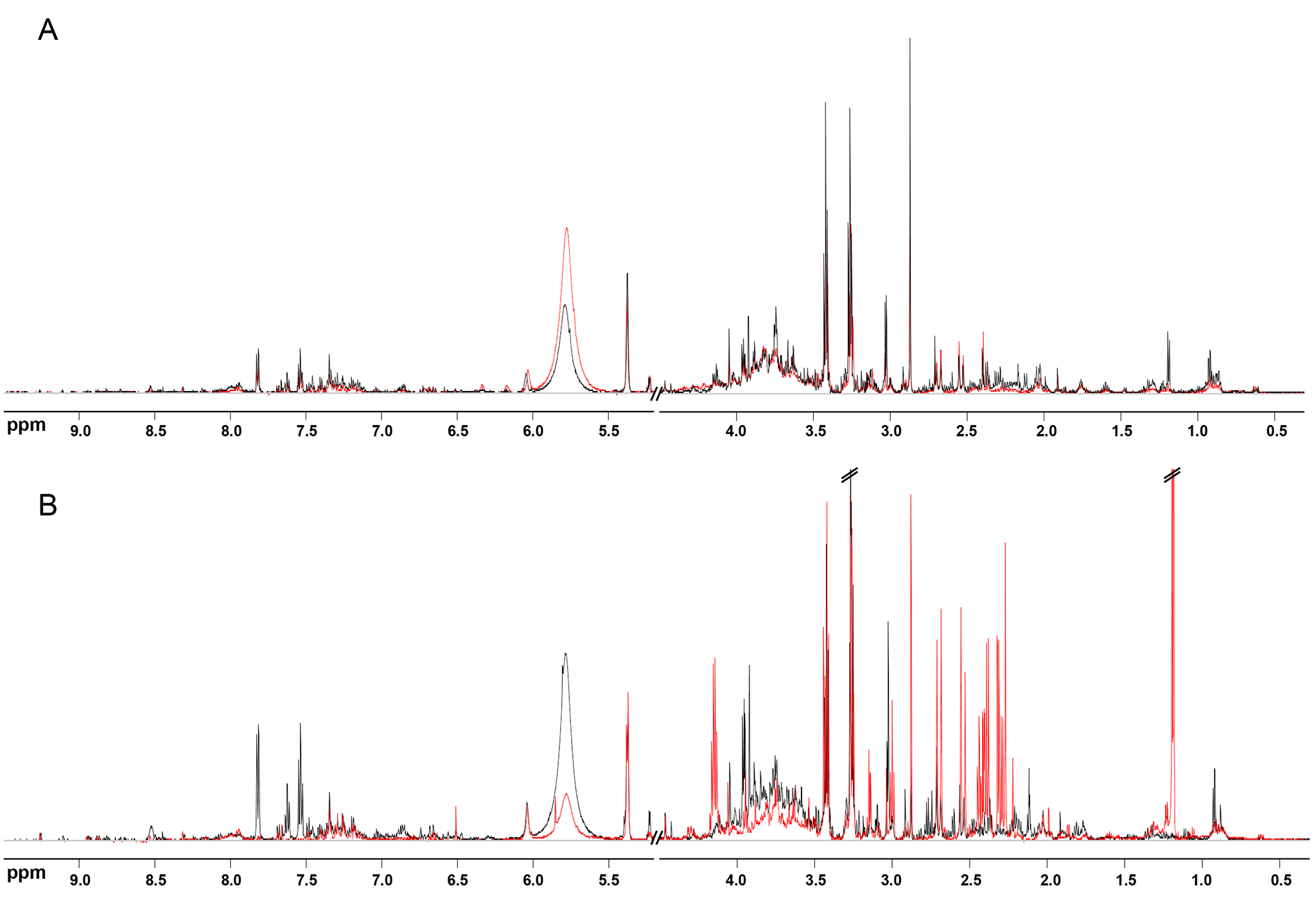

Supplement: Figure S1 — Full overlaid spectra of representative 1H NMR spectra of urine sample from lean and obese mice ( Figure 1A ). Samples from male and female mice are shown on the top and bottom, respectively. Black and red spectra are overlaid and shown for lean mice and ob/ob mice, respectively. Since there are almost no peaks in the upfield region of the spectra for serum samples and all the peaks are shown in Figure 1B, full view of the spectra are not shown here. (TIF) [file pone.0075998.s001.tif]

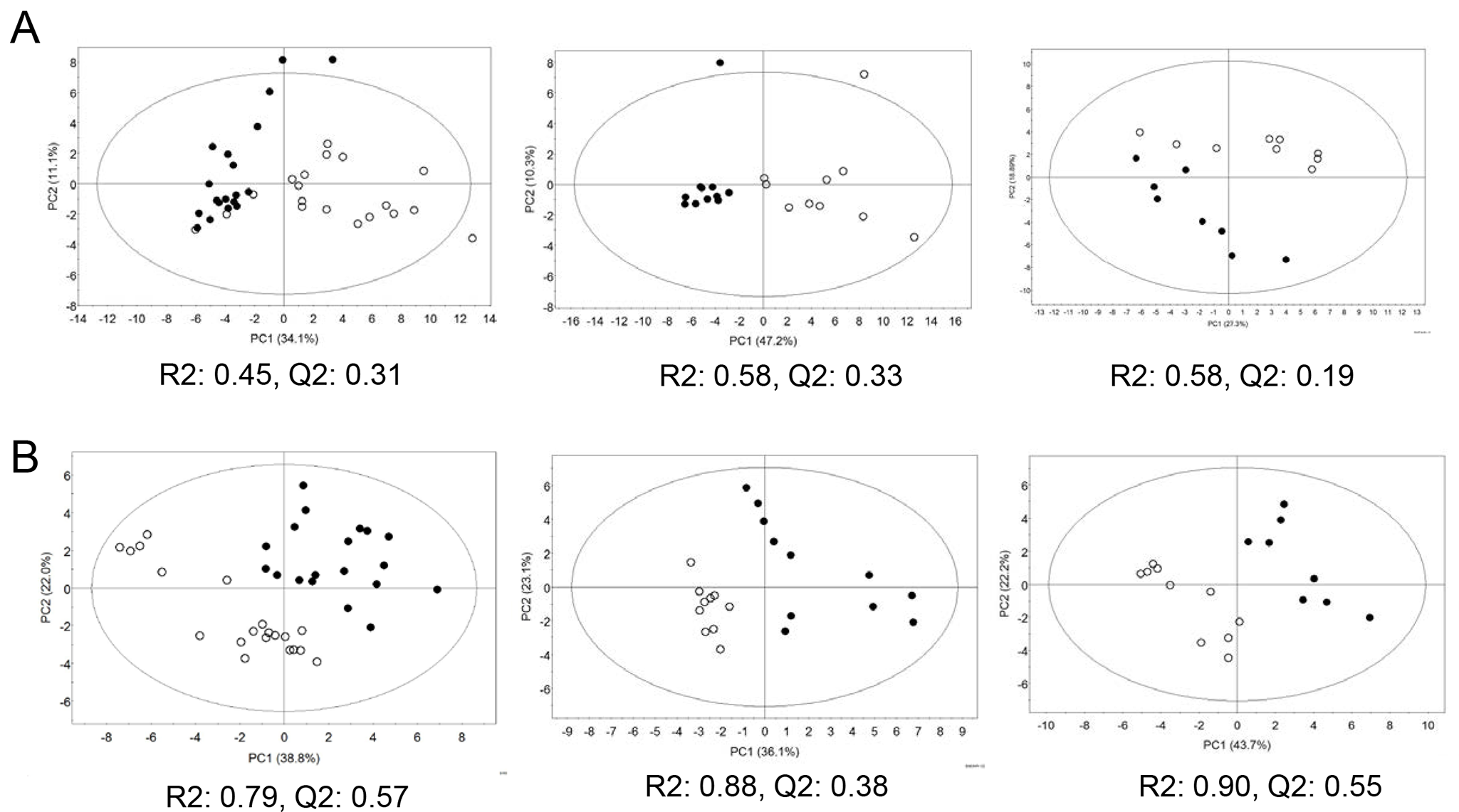

Supplement: Figure S2 — PCA score plots obtained from the 1H NMR spectra of urine (A) and serum (B) samples from lean and obese mice. All the mice including males and females, male mice, and female mice are shown on the left, middle, and right, respectively. (TIF) [file pone.0075998.s002.tif]

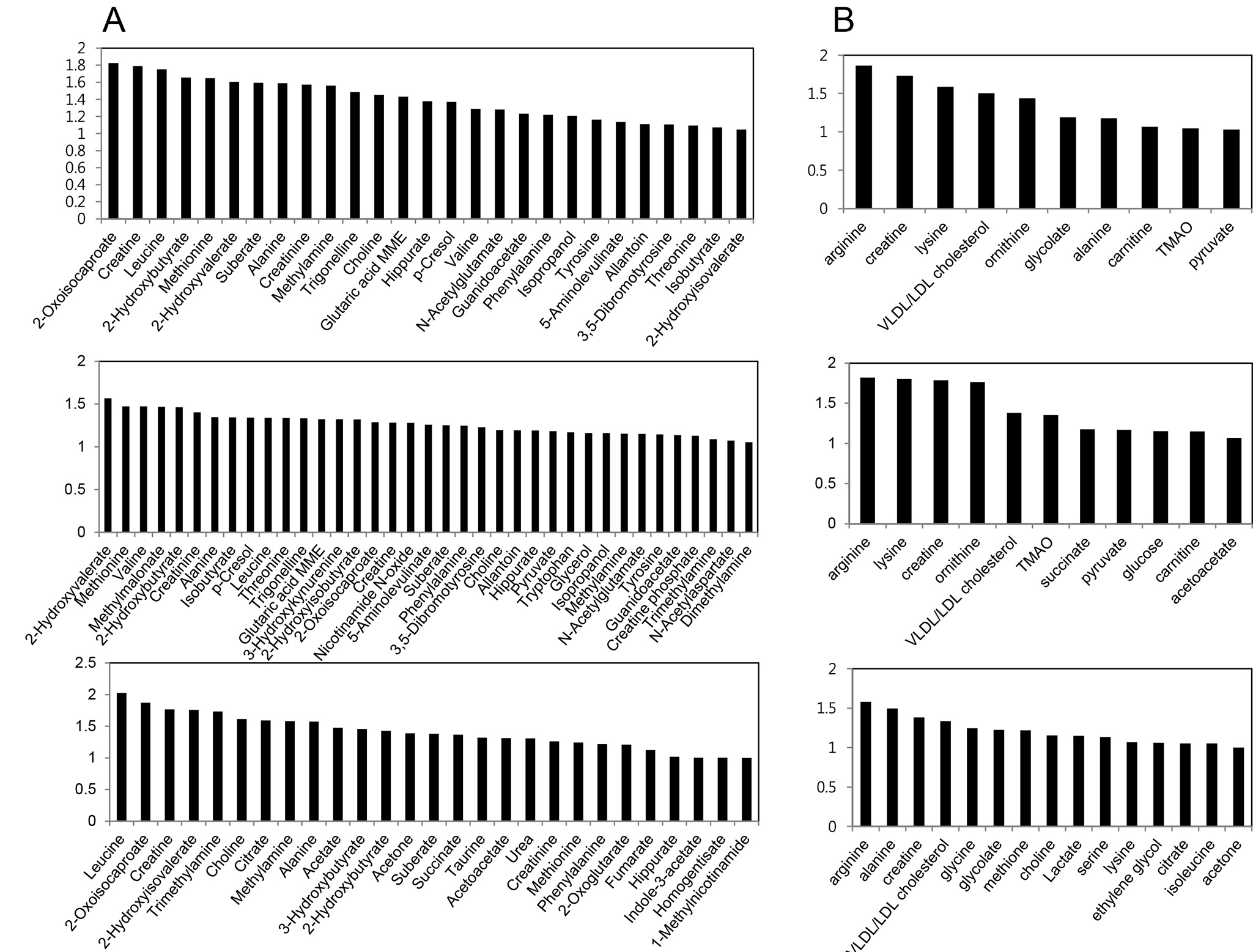

Supplement: Figure S3 — Variable importance in the projection (VIP) plots of lean vs . obese mice obtained from OPLS-DA with a threshold of 1.0. All the mice including males and females, males, and females are shown on the top, middle, and down, respectively, for urine (A) and serum (B) samples. (TIF) [file pone.0075998.s003.tif]

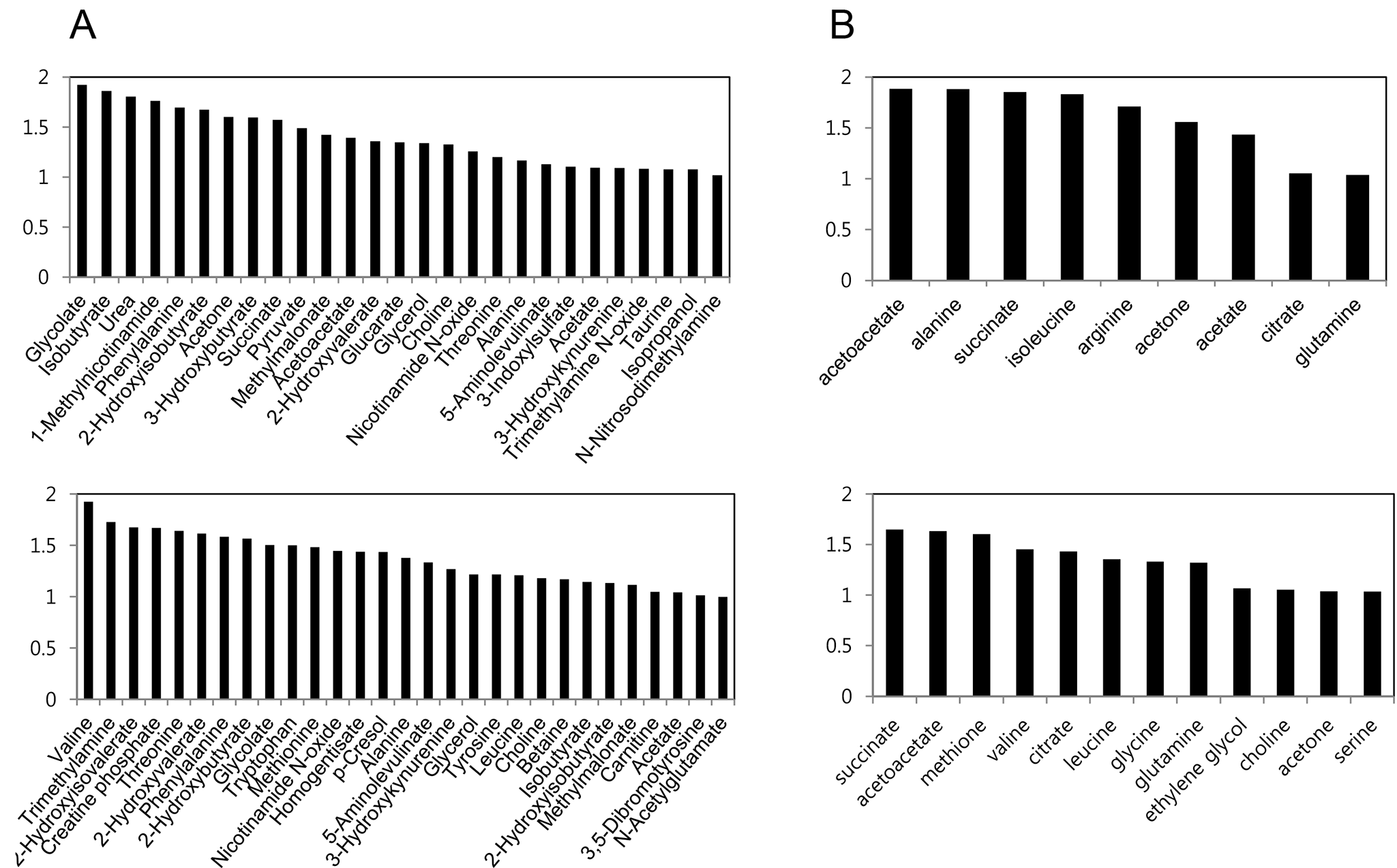

Supplement: Figure S4 — Variable importance in the projection (VIP) plots of male vs . female mice obtained from OPLS-DA with a threshold of 1.0. The urine (A) and serum (B) samples from the obese (top) and lean (down) mice are shown. (TIF) [file pone.0075998.s004.tif]

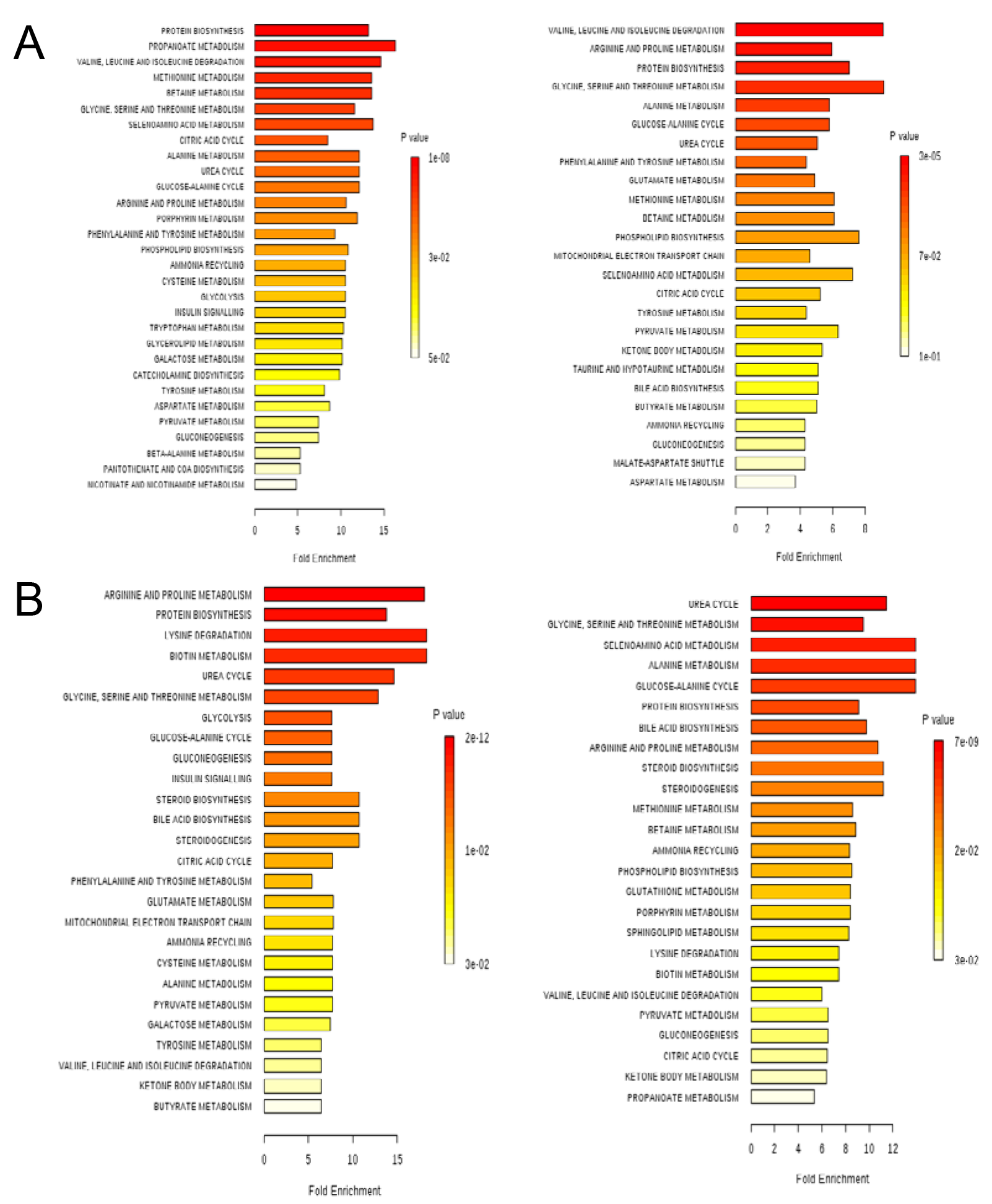

Supplement: Figure S5 — Pathway enrichment analysis of urine (A) and serum (B) samples. The 47 and 23 urine metabolites from male and female mice, respectively, and 13 and 19 serum metabolites from male and female mice, respectively, all with p<0.05 in Mann-Whitney t-tests, were used for quantitative pathway enrichment analysis. (TIF) [file pone.0075998.s005.tif]
