# Supplementary material for: Gender-Specific Metabolomic Profiling of Obesity in Leptin-Deficient ob/ob Mice by 1H NMR Spectroscopy
Source: PLoS One. 2013 Oct 3;8(10):e75998. doi: 10.1371/journal.pone.0075998 (PMC3789719; doi:10.1371/journal.pone.0075998)
Supplement: Table S1 — Summary of significantly altered urine metabolites and the related metabolic pathways in ob/ob mice in detail. (DOCX) [file pone.0075998.s006.docx]

| **Metabolite** | **CAS**  **Number** | **Direction of change** | ***p* value**^a^ | **Pathway**^b^ |
| --- | --- | --- | --- | --- |
| Guanidoacetate  Creatine  N-acetylglutamate  Creatine phosphate | 352-97-6  57-00-1  1188-37-0  67-07-2 | DOWN  DOWN  DOWN  DOWN | ***  ***  ***  * | [Arginine and proline metabolism](javascript:void(0);) |
| Choline  Creatine  Guanidoacetate  Threonine  5-aminolevulinate | 62-49-7  57-00-1  352-97-6  72-19-5  106-60-5 | DOWN  DOWN  DOWN  DOWN  DOWN | ***  ***  ***  **  ** | [Glycine, serine and threonine metabolism](javascript:void(0);) |
| 2-Hydroxybutyrate | 565-70-8 | DOWN | *** | [Propanoate metabolism](javascript:void(0);) |
| Methionine | 63-68-3 | DOWN | *** | [Cysteine and methionine metabolism](javascript:void(0);) |
| Alanine | 56-41-7 | DOWN | *** | Selenoamino acid metabolism |
| N-Acetyl aspartate  2-Oxoglutarate | 997-55-7  328-50-7 | DOWN  UP | *  * | [Alanine, aspartate and glutamate metabolism](javascript:void(0);) |
| Alanine | 56-41-7 | DOWN | *** |  |
| Alanine | 56-41-7 | DOWN | *** | [Aminoacyl-tRNA biosynthesis](javascript:void(0);) |
| Phenylalanine  Methionine  Valine  Threonine  Tyrosine  Tryptophan | 63-91-2  63-68-3  72-18-4  72-19-5  60-18-4  73-22-3 | DOWN  DOWN  DOWN  DOWN  DOWN  DOWN | ***  ***  ***  **  **  * |  |
| Choline | 62-49-7 | DOWN | *** | [Glycerophospholipid metabolism](javascript:void(0);) |
| 2-Oxoisocaproate | 328-50-7 | DOWN | *** | Valine, leucine and isoleucine degradation |
| Valine  Leucine  Acetoacetate | 72-18-4  61-90-5  541-50-4 | DOWN  DOWN  UP | ***  ***  * |  |
| Phenylalanine  Tyrosine  Tryptophan | 63-91-2  60-18-4  73-22-3 | DOWN  DOWN  DOWN | ***  **  * | [Phenylalanine, tyrosine and tryptophan biosynthesis](javascript:void(0);) |
| Phenylalanine  Tyrosine | 63-91-2  60-18-4 | DOWN  DOWN | ***  ** | [Phenylalanine metabolism](javascript:void(0);) |
| Valine  Leucine  Threonine | 72-18-4  61-90-5  72-19-5 | DOWN  DOWN  DOWN | ***  ***  ** | Valine, leucine and isoleucine biosynthesis |
| Valine | 72-18-4 | DOWN | *** | [Pantothenate and CoA biosynthesis](javascript:void(0);) |
| Tyrosine  Acetoacetate | 60-18-4  541-50-4 | DOWN  UP | **  * | [Tyrosine metabolism](javascript:void(0);) |
| Tyrosine | 60-18-4 | DOWN | ** | [Ubiquinone and other terpenoid-quinone biosynthesis](javascript:void(0);) |
| 5-Aminolevulinate | 106-60-5 | DOWN | ** | [Porphyrin and chlorophyll metabolism](javascript:void(0);) |
| Indole-3-acetate  Tryptophan  3-hydroxykynurenine | 87-51-4  73-22-3  606-14-4 | DOWN  DOWN  DOWN | *  *  * | [Tryptophan metabolism](javascript:void(0);) |
| Citrate | 77-92-9 | UP | ** | [Glyoxylate and dicarboxylate metabolism](javascript:void(0);) |
| 2-Oxoglutarate  Acetoacetate | 328-50-7  541-50-4 | UP  UP | *  * | [Butanoate metabolism](javascript:void(0);) |
| Citrate  2-Oxoglutarate | 77-92-9  328-50-7 | UP  UP | **  * | [Citric acid cycle (TCA cycle)](javascript:void(0);) |
| Glycerol | 56-81-5 | DOWN | *** | [Glycerolipid metabolism](javascript:void(0);) |
| Glycerol | 56-81-5 | DOWN | *** | [Galactose metabolism](javascript:void(0);) |
| Acetoacetate | 541-50-4 | UP | * | [Synthesis and degradation of ketone bodies](javascript:void(0);) |
| 2-Oxoglutarate | 328-50-7 | UP | * | D-glutamine and D-glutamate metabolism |
| Creatinine | 60-27-5 | DOWN | *** | Creatine metabolism |
| Creatine | 57-00-1 | DOWN | *** |  |

**Table S1. Summary of significantly altered urine metabolites and the related metabolic pathways in ob/ob mice in detail**

^a^ The p values of the individual metabolic concentration in *t*-test are shown. *, **, and *** indicate p < 0.05, p < 0.01, and p < 0.005, respectively.

^b^ The pathways were analyzed using MetaboAnalyst and concentration of metabolites as an input, and listed from low to high pathway p score. This shows no gut microbiome-derived metabolism because mouse pathway library was used for analysis.
