# Supplementary material for: Gender-Specific Metabolomic Profiling of Obesity in Leptin-Deficient ob/ob Mice by 1H NMR Spectroscopy
Source: PLoS One. 2013 Oct 3;8(10):e75998. doi: 10.1371/journal.pone.0075998 (PMC3789719; doi:10.1371/journal.pone.0075998)
Supplement: Table S2 — Summary of significantly altered serum metabolites and the related metabolic pathways in ob/ob mice in detail. (DOCX) [file pone.0075998.s007.docx]

**Table S2. Summary of significantly altered serum metabolites and the related metabolic pathways in ob/ob mice in detail**

| **Metabolite** | **CAS**  **Number** | **Direction of change** | ***p* value**^a^ | **Pathway**^b^ |
| --- | --- | --- | --- | --- |
| Arginine  Creatine  Ornithine | 74-79-3  57-00-1  70-26-8 | DOWN  DOWN  DOWN | ***  ***  *** | [Arginine and proline metabolism](javascript:void(0);) |
| Lysine | 56-87-1 | DOWN | *** | [Lysine biosynthesis](javascript:void(0);) |
| Lysine | 56-87-1 | DOWN | *** | [Biotin metabolism](javascript:void(0);) |
| Lysine  Carnitine | 56-87-1  406-76-8 | DOWN  UP | ***  *** | [Lysine degradation](javascript:void(0);) |
| Alanine  Lysine  Arginine  Serine  Phenylalanine  Glycine  Methionine  Isoleucine | 56-41-7 | DOWN | *** | [Aminoacyl-tRNA biosynthesis](javascript:void(0);) |
|  | 56-87-1  74-79-3  56-45-1  63-91-2  56-40-6  63-68-3  73-32-5 | DOWN  DOWN  DOWN  DOWN  DOWN  DOWN  DOWN | ***  ***  **  **  *  *  * |  |
| Cholesterol | 57-88-5 | UP | *** | [Steroid](javascript:void(0);) biosynthesis |
| Cholesterol | 57-88-5 | UP | *** | Steroid hormone biosynthesis |
| Cholesterol  Glycine | 57-88-5  56-40-6 | UP  DOWN | ***  * | Primary bile acid biosynthesis |
| Orinithine  Glycine | 70-26-8  56-40-6 | DOWN  DOWN | ***  * | [Glutathione metabolism](javascript:void(0);) |
| Creatine  Pyruvate  Choline  Serine  Glycine | 57-00-1  127-17-3  62-49-7  56-45-1  56-40-6 | DOWN  DOWN  DOWN  DOWN  DOWN | ***  ***  **  **  * | [Glycine, serine and threonine metabolism](javascript:void(0);) |
| Pyruvate | 127-17-3 | DOWN | *** | [Alanine, aspartate and glutamate metabolism](javascript:void(0);) |
| Alanine | 56-41-7 | DOWN | *** |  |
| Alanine | 56-41-7 | DOWN | *** | Selenoamino acid metabolism |
| Pyruvate  Acetoacetate | 127-17-3  541-50-4 | DOWN  UP | ***  * | [Butanoate metabolism](javascript:void(0);) |
| Pyruvate  Isoleucine | 127-17-3  73-32-5 | DOWN  DOWN | ***  * | [Valine, leucine and isoleucine biosynthesis](javascript:void(0);) |
| Pyruvate  Serine  Methionine | 127-17-3  56-45-1  63-68-3 | DOWN  DOWN  DOWN | ***  **  * | [Cysteine and methionine metabolism](javascript:void(0);) |
| Glycolate  Citrate | 79-14-1  77-92-9 | DOWN  DOWN | ***  * | [Glyoxylate and dicarboxylate metabolism](javascript:void(0);) |
| Serine | 56-45-1 | DOWN | ** | [Sphingolipid metabolism](javascript:void(0);) |
| Pyruvate  Lactate | 127-17-3  79-33-4 | DOWN  DOWN | ***  * | Pyruvate metabolism |
| Pyruvate  Lactate | 127-17-3  79-33-4 | DOWN  DOWN | ***  * | [Glycolysis or Gluconeogenesis](javascript:void(0);), |
| Pyruvate  Citrate | 127-17-3  77-92-9 | DOWN  DOWN | ***  * | [Citrate cycle (TCA cycle)](javascript:void(0);) |
| Choline | 62-49-7 | DOWN | ** | [Glycerophospholipid metabolism](javascript:void(0);) |
| Serine  Glycine | 56-45-1  56-40-6 | DOWN  DOWN | **  * | Methane metabolism |
| Serine  Glycine | 56-45-1  56-40-6 | DOWN  DOWN | **  * | [Cyanoamino acid metabolism](javascript:void(0);) |
| Phenylalanine | 63-91-2 | DOWN | ** | Phenylalanine, tyrosine and tryptophan biosynthesis |
| Phenylalanine | 63-91-2 | DOWN | ** | Phenylalanine metabolism |
| Isoleucine  Acetoacetate | 73-32-5  541-50-4 | DOWN  UP | *  * | [Valine, leucine and isoleucine degradation](javascript:void(0);) |
| 4-hydroxyphenylacetate  Acetoacetate | 156-38-7  541-50-4 | DOWN  UP | *  * | [Tyrosine metabolism](javascript:void(0);) |
| Glycine | 56-40-6 | DOWN | * | [Porphyrin and chlorophyll metabolism](javascript:void(0);), |
| Glycine | 56-40-6 | DOWN | * | [Nitrogen metabolism](javascript:void(0);), |
| Acetoacetate | 541-50-4 | UP | * | [Synthesis and degradation of ketone bodies](javascript:void(0);) |

^a^ The p values of the individual metabolic concentration in *t*-test are shown. *, **, and *** indicate p < 0.05, p < 0.01, and p < 0.005, respectively.

^b^ The pathways are analyzed using MetaboAnalyst and concentration of metabolites as an input, and listed from low to high pathway p score. This shows no gut microbiome-derived metabolism because mouse pathway library was used for analysis.
